# Competing risks to breast cancer mortality in Catalonia

**DOI:** 10.1186/1471-2407-8-331

**Published:** 2008-11-12

**Authors:** Ester Vilaprinyo, Rosa Gispert, Montserrat Martínez-Alonso, Misericòrdia Carles, Roger Pla, Josep-Alfons Espinàs, Montserrat Rué

**Affiliations:** 1 Institut d'Investigació Biomèdica de Bellvitge, IDIBELL, Hospitalet de Llobregat Catalonia, Spain; 2 Servei d'Informació i Estudis, Departament de Salut, Generalitat de Catalunya, Catalonia, Spain; 3 Institut de Recerca Biomèdica de Lleida (IRBLLEIDA)-Universitat de Lleida, Catalonia, Spain; 4 Facultat de Ciències Econòmiques i Empresarials, Universitat Rovira i Virgili, Catalonia, Spain; 5 Institut Català de la Salut a Terres de l'Ebre, Tortosa, Catalonia, Spain; 6 Pla Director d'Oncologia, Departament de Salut, Generalitat de Catalunya, Catalonia, Spain

## Abstract

**Background:**

Breast cancer mortality has experienced important changes over the last century. Breast cancer occurs in the presence of other competing risks which can influence breast cancer incidence and mortality trends. The aim of the present work is: 1) to assess the impact of breast cancer deaths among mortality from all causes in Catalonia (Spain), by age and birth cohort and 2) to estimate the risk of death from other causes than breast cancer, one of the inputs needed to model breast cancer mortality reduction due to screening or therapeutic interventions.

**Methods:**

The multi-decrement life table methodology was used. First, all-cause mortality probabilities were obtained by age and cohort. Then mortality probability for breast cancer was subtracted from the all-cause mortality probabilities to obtain cohort life tables for causes other than breast cancer. These life tables, on one hand, provide an estimate of the risk of dying from competing risks, and on the other hand, permit to assess the impact of breast cancer deaths on all-cause mortality using the ratio of the probability of death for causes other than breast cancer by the all-cause probability of death.

**Results:**

There was an increasing impact of breast cancer on mortality in the first part of the 20^th ^century, with a peak for cohorts born in 1945–54 in the 40–49 age groups (for which approximately 24% of mortality was due to breast cancer). Even though for cohorts born after 1955 there was only information for women under 50, it is also important to note that the impact of breast cancer on all-cause mortality decreased for those cohorts.

**Conclusion:**

We have quantified the effect of removing breast cancer mortality in different age groups and birth cohorts. Our results are consistent with US findings. We also have obtained an estimate of the risk of dying from competing-causes mortality, which will be used in the assessment of the effect of mammography screening on breast cancer mortality in Catalonia.

## Background

Breast cancer is the leading cause of mortality among middle-aged women in many developed countries, including Catalonia, a region in the northeast of Spain, where it accounts for a fifth of all female cancer deaths and, on the average, fourteen years of potential life lost per death from this cause [1].

Causes of death other than breast cancer may influence mortality trends in two ways: 1) by changing the number of women at risk of having breast cancer and, 2) by changing the risk of dying of breast cancer once it has developed. For example, infectious diseases at the beginning of the 20^th ^century reduced the number of women at risk for breast cancer and also competed with breast cancer as a cause of death in women with this disease. Thus, the effect of other causes of death in breast cancer incidence and mortality has changed over time depending on trends in other competing risks.

Rosenberg [2] used the multi-decrement life table methodology to partition overall mortality into mortality due to breast cancer and mortality due to other causes. In addition, multi-decrement life tables permit assessment of the impact that breast cancer mortality has on overall mortality by birth cohort and age. For instance, in the US, the reduction in overall mortality when removing breast cancer as a cause of death could be as high as 15% at some ages.

Evaluation of the impact of mammography and adjuvant therapy on breast cancer mortality reduction can be done using statistical models, for example those found in several studies sponsored by CISNET [3]. The modeling process requires information such as the dissemination of mammography and adjuvant treatment programs, diagnostic characteristics of mammography, breast cancer incidence and mortality. Another required input is the competing-cause mortality, taking into account that deaths due to breast cancer occur in the presence of other causes of death, the above-mentioned competing risks.

The aims of the present work are: 1) to assess the impact of breast cancer mortality on overall mortality by birth cohort and age in Catalonia in the 20^th ^century and 2) to assess the risk of death from other causes than breast cancer in cohorts born from 1900 to 2004. Our analysis is based on Rosenberg's work for the US and is part of a wider project that aims to model the impact of mammography in Catalonia (Spain) based on the methodology developed by Lee and Zelen for the CISNET project [4].

## Methods

### Data Sources

Catalonia is an autonomous region of Spain which has had authority over health-care planning, administration and provision since 1985. It has approximately one sixth of the Spanish population. The Catalan Health Department has implemented an independent information system and preventive programs in the region. Population breast cancer screening programs were initiated in the early nineties [5].

By the year 2007, the Catalan Health Service was providing services to 7 million inhabitants, including 3.5 million women. The female population grew from 800.000 women in 1900 to more than 3 million in 2004. The number of breast cancer deaths increased from an average of 612 per year in the period 1975–79 to 1060 per year in the period 1990–94 and then decreased to an average of 1000 per year in the period 2000–04. For the same periods, the percentage of breast cancer deaths among overall mortality in women increased from 3.5% to 5.2% and then decreased to 3.6%.

Table 1 shows the data sources used in this study and describes their characteristics. The National Institute of Statistics (INE [6]) and the Catalan Institute of Statistics (IDESCAT [7]) have provided estimations of annual population changes since 1970. These changes were estimated by the component method [8] which takes into account vital statistics (births and deaths) and immigration to adjust official post-census estimates. We obtained the mid-year population data using linear interpolation. Mortality data has been officially collected and registered by the Catalan Mortality Registry [9] and the INE. These two institutions had this responsibility during different periods (see Table 1). Population and deaths correspond to residents in Catalonia. All data are public and available from their annual reports and web pages [6,7,9].

**Table 1:** Description and sources of data.

**Data**	**Period**	**Description**	**Source**
**Population**	1900–1970	Official census data (every 10 years)	National Institute of Statistics (INE)
	1970–1985	Annual population estimated by the components method*	INE
	1986–2004	Annual population estimated by the components method*	Catalan Institute of Statistics (IDESCAT)

**All-cause mortality**	1900–1974	Annual counts	INE
	1975–2004	Annual counts	Catalan Mortality Registry

**Breast cancer mortality**	1975–2004	Annual counts	Catalan Mortality Registry

*For a detailed description of the component method see reference [8]

We grouped cohorts and ages into five-year categories in order to smooth data and minimize fluctuations present in some census and mortality data prior to 1960 [10], and to get more reliable estimates for young age groups. We used linear interpolation to estimate missing data or to split categories that were larger than five years in the original data source.

### Statistical analysis

Deaths by calendar year were converted into deaths by year of birth (cohorts) using the relation *year of birth = calendar year of death – age at death*.

Actuarial life table methods were used to obtain the life table functions: the age specific death rate _*n*_*M*_*x*,*BY*_, the conditional probabilities of death _*n*_*q*_*x*,*BY*_, and the proportion of expected survivors by age *l*_*x*,*BY *_[11]. These functions were estimated for a) the overall population, and b) for a population in which breast cancer had been eliminated as a cause of death, in the following steps:

1) We obtained age-specific death rates, _*n*_*M*_*x*,*BY*_, for age groups [*x*, *x+n*) and birth cohorts *BY*, using the expression:

 nMx,BY= nDx,BY nKx,BY

where _*n*_*D*_*x*,*BY *_is the observed number of deaths and _*n*_*K*_*x*,*BY *_the midyear population in age group *x *to *x+n*. In our analysis *n *is equal to 5 years.

2) We estimated overall probabilities of death _*n*_*q*_*x*,*BY *_in the interval [*x*, *x+n*) with the formula:

 nqx,BY= nMx,BY1n[1+n(1− nfx) nMx,BY]

where _*n*_*f*_*x *_is the estimated fraction of years lived in the interval. Assuming a uniform distribution of deaths over time we have taken _*n*_*f*_*x *_= 0.5 for all age groups except the 0–4 age group, where _*n*_*f*_0 _= 0.4. All-cause probabilities of death are labeled as  nqx,BYτ.

3) We estimated probabilities of death from breast cancer, $q_{x,BY}^{bc}$, in the interval [*x, x+n*) as described in Step 2, using only breast cancer deaths in the numerator.

4) Probabilities of death in the last age group, 85+, were estimated as  ∞q85,BYτ = 1 for overall mortality, and  ∞q85,BYbc= ∞D85,BYbc/ ∞K85,BY for breast cancer mortality.

5) We estimated the missing breast cancer mortality probabilities for earlier years of birth using an age-cohort model. The observed breast cancer death rates were used to fit the following quasi-Poisson model that provides maximum likelihood estimates of mortality rates (_*n*_*M*_*x*,*BY*_) based on age (*x*) and year of birth (*BY*):

 nMx,BY=e(β0+βage1x+βage2x2+βage3x3+βage4x4+βcohort1BY+βcohort2BY2)

**Figure 1:**
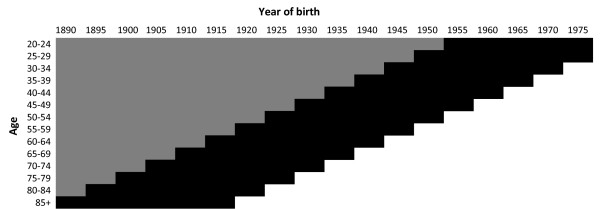
**Breast cancer probabilities of death by age and year of birth, Catalonia (Spain).** The black cells indicate the available data. The grey cells indicate data that are estimated using an age-cohort model. Years of birth are grouped in five-year intervals and are labeled with the first year of the interval.

The age-cohort model parameters estimated using the generalized linear models package of the R software [12] were: β_0_: -1.59 × 10^3^, β_age1_: 1.05, β_age2_: -2.06 × 10^-2^, β_age3_: 1.75 × 10^-4^, β_age4_: -5.21 × 10^-7^, β_cohort1_: 1.62, and β_cohort2_: -4.2 × 10^-4^. Then the probabilities were estimated as described in Step 2. Figure 1 shows the periods and age groups where breast cancer mortality data was available (in black) or estimated (in gray).

6) We subtracted the probabilities of dying from breast cancer from the overall probabilities of death to obtain the probabilities of dying from causes other than breast cancer  nqx,BY−bc.

 nqx,BY−bc= nqx,BYτ− nqx,BYbc

7) We obtained the life table function *l*_*x*,*BY*_, which indicates the proportion of survivors at age *x *from birth cohort *BY*, using the expression:

*l*_*x*+*n*,*BY *_= *l*_*x*,*BY *_(1 - _*n*_*q*_*x*,*BY*_), starting from *l*_0,*BY *_= 1

Following the same nomenclature used in probabilities of death, the functions $l_{x,BY}^{\tau }$ and $l_{x,BY}^{-bc}$ were computed. The $l_{x,BY}^{-bc}$ indicates the proportion of survivors from birth cohort *BY*, at the beginning of each age interval, after removing breast cancer as a cause of death.

8) We computed the ratio of  nqx,BY−bc/ nqx,BYτ, which measures the impact in all-cause mortality of removing deaths from breast cancer at different birth cohorts and ages. A ratio equal to 1, in a specific group, would indicate that there is no breast cancer mortality in that group. A ratio equal to 0 would indicate that all mortality is due to breast cancer.

## Results

### Breast cancer impact on overall mortality

Cohort multi-decrement life table functions $q_{x,BY}^{-bc}$ and $l_{x,BY}^{-bc}$ were obtained for a population of women born between 1900 and 2004, with breast cancer removed as a cause of death.

**Figure 2:**
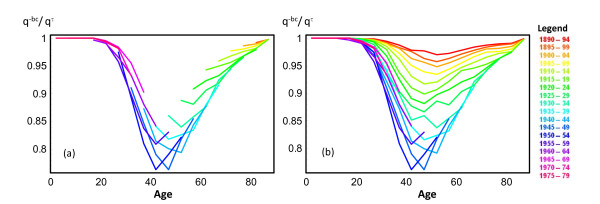
**Ratio of the probability of death with breast cancer removed to the probability of all-cause mortality ($q_{x}^{-bc}/q_{x}^{\tau }$), by age and year of birth**. (a) and (b) show results for available and for estimated data, respectively.

Figure 2 presents the ratio  nqx,BY−bc/ nqx,BYτ by age and cohort of birth. This ratio can be interpreted as the proportion of deaths attributable to causes other than breast cancer. It facilitates evaluating the impact of removing breast cancer as a cause of death. Figures 2a and 2b show the ratio before and after the extrapolation of breast cancer probabilities of death, respectively.

**Figure 3:**
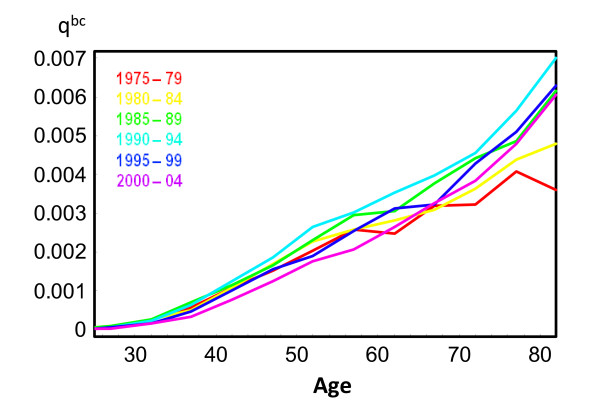
**Probabilities of breast cancer death (**$q_{x}^{bc}$**) for selected calendar years.**

**Figure 4:**
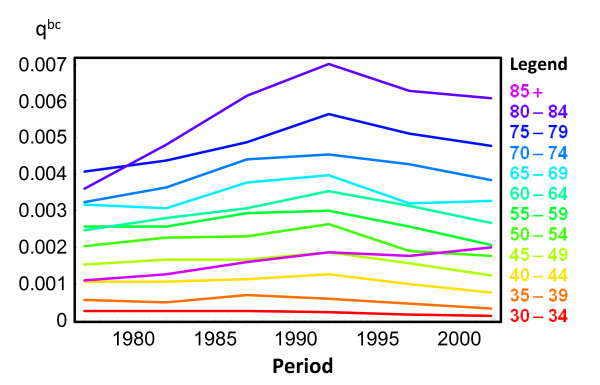
**Probabilities of breast cancer death (**$q_{x}^{bc}$**) by age.**

The impact of removing breast cancer as a cause of death is small under age 30 and over age 70. Below age 30 the number of breast cancer cases is small and above age 70 mortality from other causes, such as cardiovascular diseases, increases. The largest impact of removing breast cancer as a cause of death in overall mortality is observed at ages 40–54. For specific birth cohorts, the largest observed impact was in the 40–44 age group for cohorts born in 1945–49 and the 45–49 age group for cohorts born in 1950–54 (24% of all-cause mortality for both cohorts). Impacts assessed using observed data for cohorts born after 1955 tended to decrease. This finding is further supported by Figure 3, which shows the trend of breast cancer mortality by age for five year periods from 1975–79 to 2000–04. Breast cancer probability of death increased in all age groups from 1975–79 to 1990–94. In contrast, the last two studied periods, 1995–99 and 2000–04, show a reduction in breast cancer risk of death. Furthermore, Figure 4 shows how breast cancer mortality probabilities increased until the early 1990s, and then began to decrease, in all age groups.

### Risk of death from other causes than breast cancer

The $q_{x,BY}^{-bc}$ function estimates the risk of death from other causes when breast cancer is eliminated. The *l*_*x*,*BY *_function denotes the expected number of survivors at age *x*. The $l_{x,BY}^{-bc}$ values can be used as an estimation of the proportion of survivors, up to age *x*, if breast cancer was eliminated as a cause of death. As an illustrative example, Table 2 shows the values of $l_{x}^{\tau }$ and $l_{x,BY}^{-bc}$ for cohorts born in the years 1920–24, 1930–34, and 1940–44. As expected, the $l_{x}^{\tau }$ data show an increasing trend in the probability of surviving to a specific age by birth cohort. For instance, the proportion of survivors at age 20 was 75% for women born in the early 1920s, and increased to 90% for women born in the early 1940s.

**Table 2:** Proportion of survivors, $l_{x}^{\tau }$, and proportion of survivors after removing breast cancer as a cause of death, $l_{x}^{-bc}$, by age *x *and cohort of birth.

Ages	Year of Birth								
	
	1920–24			1930–34			1940–44		
	
	$l_{x}^{\tau }$	$l_{x}^{-bc}$	Dif.*	$l_{x}^{\tau }$	$l_{x}^{-bc}$	Dif.*	$l_{x}^{\tau }$	$l_{x}^{-bc}$	Dif.*
20–24	0.7474	0.7474	0	0.8499	0.8499	0	0.8977	0.8977	0
25–29	0.7338	0.7338	0.14	0.8435	0.8435	0.17	0.8953	0.8953	0.17
30–34	0.7229	0.7230	0.83	0.8389	0.8390	0.97	0.8927	0.8928	0.97
35–39	0.7162	0.7165	3.00	0.8346	0.8350	3.56	0.8894	0.8897	3.20
40–44	0.7096	0.7104	7.91	0.8288	0.8297	9.42	0.8854	0.8862	8.20
45–49	0.7017	0.7034	16.45	0.8218	0.8236	17.74	0.8803	0.8821	17.65
50–54	0.6913	0.6941	28.61	0.8129	0.8159	30.16	0.8730	0.8762	32.23
55–59	0.6760	0.6800	39.65	0.8016	0.8064	48.20	0.8620	0.8675	54.90
60–64	0.6574	0.6630	56.01	0.7853	0.7924	70.91	0.8481	0.8557	76.17
65–69	0.6342	0.6414	72.63	0.7634	0.7731	96.93	-	-	-
70–74	0.6001	0.6094	93.00	0.7320	0.7438	117.75	-	-	-
75–79	0.5499	0.5612	112.95	-	-	-	-	-	-
80–84	0.4733	0.4859	125.85	-	-	-	-	-	-
85+	-	-	-	-	-	-	-	-	-

Note: $l_{x}^{-bc}$ can be interpreted as the proportion of women at risk of dying from competing risks at age *x*.

* Indicates the number of women saved up to age *x *from an initial cohort of 10,000 women, if breast cancer was removed as a cause of death.

The column *Dif*. in Table 2 contains the difference $l_{x,BY}^{-bc}-l_{x,BY}^{\tau }$ per 10,000 women. It can be interpreted as the number of women saved up to age *x *from an initial cohort of 10,000 women, when breast cancer is removed as a cause of death. If there were no breast cancer deaths, for each 10,000 women born in 1920–24 approximately 93 would have been saved before arriving at age 70, and 118 women for the cohort born in 1930–34.

Figure 2 and Table 2 show two different approaches to evaluating trends in breast cancer mortality in the presence of other causes of death by age and cohort. The $q_{x,BY}^{-bc}$ estimates competing-cause mortality, the ratio  nqx,BY−bc/ nqx,BYτ shows the impact of breast cancer over global mortality, and $l_{x,BY}^{\tau }-l_{x,BY}^{-bc}$ indicates the number of women saved up to age *x *if breast cancer was removed as a cause of death.

## Discussion and conclusion

Our study shows that breast cancer mortality in Catalonia has experienced important changes over the last century. There was an increasing impact of breast cancer on overall mortality in the first part of the century, with a peak for cohorts born in 1945–54 in the 40–49 age groups (approximately 24% of mortality was due to breast cancer in these age groups and cohorts). This increasing impact could be explained by a decrease in mortality from other causes of death and increased breast cancer incidence and mortality due to changes in lifestyle and reproductive patterns [13]. In Catalonia, mortality from all causes in women has been decreasing an average of 1.6% per year since 1978 [14], whereas breast cancer incidence increased 2.2% per year between 1980–97 [15].

The subsequent decrease in impact was due, primarily, to the observed reduction in mortality from breast cancer during the 1990s, which affected all age groups (see [16] and official reports by the Catalan Department of Health [14]). Furthermore, the significant reduction in recent decades implies important gains in life expectancy for middle-aged women [17]. This reduction has been attributed to the use of mammography and adjuvant treatments in the US [18]. The phenomenon is now being studied in Catalonia.

In Catalonia, as in the US, breast cancer mortality had the greatest impact on global mortality in the 40–54 age groups during the 20^th ^century [2]. On one hand, breast cancer incidence and mortality are low before the age of 40 [19]. Although breast cancer incidence and mortality increase with age, after age 50 there are other causes of death (competing risks) that are acting simultaneously, and therefore breast cancer may have less impact on overall mortality.

It is worthwhile to note that the values of the ratio  nqx,BY−bc/ nqx,BYτ were higher in the US, which means that the impact of breast cancer mortality was lower. The ratio  nqx,BY−bc/ nqx,BYτ depends on two risks, the risk of dying of causes other than breast cancer (numerator) and the risk of dying of any cause (denominator). For cohorts of women born in the middle of the 20^th ^century, overall mortality rates at ages 40 to 54 years were 40% to 60% higher in the US than in Catalonia [20]. On the other hand, breast cancer mortality rates for these ages and cohorts were similar or slightly higher in Spain than the US. Since breast cancer mortality rates represent about one fifth of the mortality in these age groups, differences in the overall mortality risk in the US and Catalonia may explain the differences seen in the  nqx,BY−bc/ nqx,BYτ ratio. But, other factors like reproductive patterns, hormonal replacement therapy use and fat intake could explain differences in the impact of breast cancer mortality in both countries.

Results from Rosenberg show that the lowest  nqx,BY−bc/ nqx,BYτ ratio (the highest impact of breast cancer mortality) for women born in 1930 occurs at older ages than for those born in 1950. This pattern is also shown in our data before any extrapolation (Figure 2a). In Catalonia this is related with a clear period effect during the 1990s. As we show in Figures 3 and 4, trends for breast cancer death probabilities by age increased from the 1970s to the 1990s and started a decreasing trend after that for all age groups.

The declines in mortality from breast cancer, which began in Europe in the late 1980s may be attributed in part to earlier detection by screening programs. But, since the declining trends started before screening was introduced and occurred also in non-screened age groups, improved cancer treatments such as adjuvant chemotherapy and tamoxifen may have been important determinants of breast cancer mortality reduction [21]. In Catalonia, breast cancer mortality started to decrease at the beginning of the 1990s, concurrent with the dissemination of mammography. Since the effect of screening would be seen some years later, treatments probably had an important role during the 1990s. The contribution of each of these factors still needs to be determined in Catalonia.

Our work was undertaken in the Catalan region and not in the whole country of Spain. This is due to the fact that there is no national cancer registry in Spain. Instead, there are 12 local cancer registries, which show differences in the incidence of breast cancer by region (probably due to different reproductive patterns and degrees of economic development). The only statistics that are collected in a standardized manner at the national level are mortality data. Also, this study is part of a project that aims to assessing the cost-effectiveness of different early detection strategies on the reduction of breast cancer mortality. The cost-effectiveness analysis needs information on outcomes and costs that would be difficult to obtain at the national level. On the other hand, since Catalonia has approximately one sixth of the Spanish population, we believe that some of the results of our studies will be relevant for all of Spain.

This study has several limitations. First, the multi-decrement method that we used assumes independence of causes of death, which means that when breast cancer mortality is removed, the risk of dying due to the remaining causes of death is not affected. This assumption may not be true, since different causes of death may share the same risk factors. For example, body weight, smoking, and diet are associated not only with breast cancer but also with other health problems, like cardiovascular diseases. Therefore, the probability of dying of other causes could change when breast cancer death is removed. In our study, if mortality from other causes had decreased when eliminating breast cancer mortality, the impact of breast cancer mortality would be higher than the reported values. There are methods for competing risks analysis, such as cumulative incidence functions, that do not make any assumptions about independence of risks, but need information on the relationships among them [22,23]. These methods are more complex than multi-decrement life tables and have been used to evaluate the effects of explanatory variables, such as assessing the effects of therapy in different groups with multiple endpoints. On the other hand, Chin Long Chiang proposed a method [24] that takes into account different conditional probabilities of death (crude, net and partial crude) which reflect the relationships among the different risks of death acting simultaneously. Nevertheless, although these methods could be more accurate than the one used in our work, they make assumptions about the risks' associations requiring information that was not available to us such as causes of death for women with breast cancer. Therefore, we chose the multi-decrement method, that Rosenberg also used [2], because it is simple and adequate to fulfill our objectives.

A second limitation concerns quality of information. The validity of population and mortality data has increased over time, but there may be errors in data from the earliest years of the study. We observed some fluctuations in the late 1930s and early 1940s, coinciding with the Spanish Civil War, a problem that could be addressed by smoothing the life table series' before using them in subsequent analyses. Also, to minimize variability we aggregated data in 5-year age groups and calendar years. The quality of mortality statistics has also changed over time, but different studies have shown that in Spain, deaths from cancer as a whole and leading cancer sites (lung, colon-rectum, prostate, stomach, pancreas, female breast, uterus, brain, leukemia, lymphomas and myeloma) were properly coded [25,26]. Furthermore, breast cancer mortality data was provided by the Catalan Mortality Registry which, since the beginning of the 1980s, collects data and codes causes of death consistently and has adequate quality standards.

A third limitation arises from the assumptions made when completing information on breast cancer mortality probabilities for years without available data. Data from available years seemed adequate to us to fit an age-cohort model that predicted missing information. Other approximations such as the assumption that the probabilities of dying from breast cancer at a given age would be the same in neighboring years with data, as Rosenberg did, do not change the main results (data not shown). In any case, extrapolated data for the earlier cohorts may differ from the actual values and therefore results based on these data should be interpreted with caution.

In summary, we obtained cohort life tables for causes of death other than breast cancer in Catalonia (Spain). This makes it possible to quantify the impact of removing breast cancer on overall mortality in different age groups and birth cohorts ( nqx,BY−bc/ nqx,BYτ). As with the US results, our analysis found that the greatest impact of breast cancer mortality on overall mortality was for women aged 40–54 born in the middle of the 20^th ^century. The multi-decrement life tables method also provided estimations of the risk of dying from competing risks that will be used in the assessment of the effect of mammography screening on breast cancer mortality.

## Competing interests

The authors declare that they have no competing interests.

## Authors' contributions

MR, EV, MC, RP and JAE participated in design and coordination of the study. EV and MR performed the statistical analysis and drafted the manuscript. RG provided mortality data and contributed to the interpretation of results. MM developed the age-cohort model for breast cancer mortality. All authors read and approved the final manuscript.

## Pre-publication history

The pre-publication history for this paper can be accessed here:


